# Supplementary material for: Phenotypic divergence between the cultivated apple (Malus domestica) and its primary wild progenitor (Malus sieversii)
Source: PLoS One. 2022 Mar 23;17(3):e0250751. doi: 10.1371/journal.pone.0250751 (PMC8942233; doi:10.1371/journal.pone.0250751)
Supplement: S1 Fig — Phenotypes include acidity change during storage, acidity, precocity, harvest date, firmness, and weight. Cultivated apple scores for each phenotype are shown in blue, and the ancestral state of each phenotype is represented in yellow as a density distribution of values from M. sieversii. The R and p values from a Pearson correlation between phenotypic values and release year are shown within each scatter plot. (DOCX) [file pone.0250751.s001.docx]

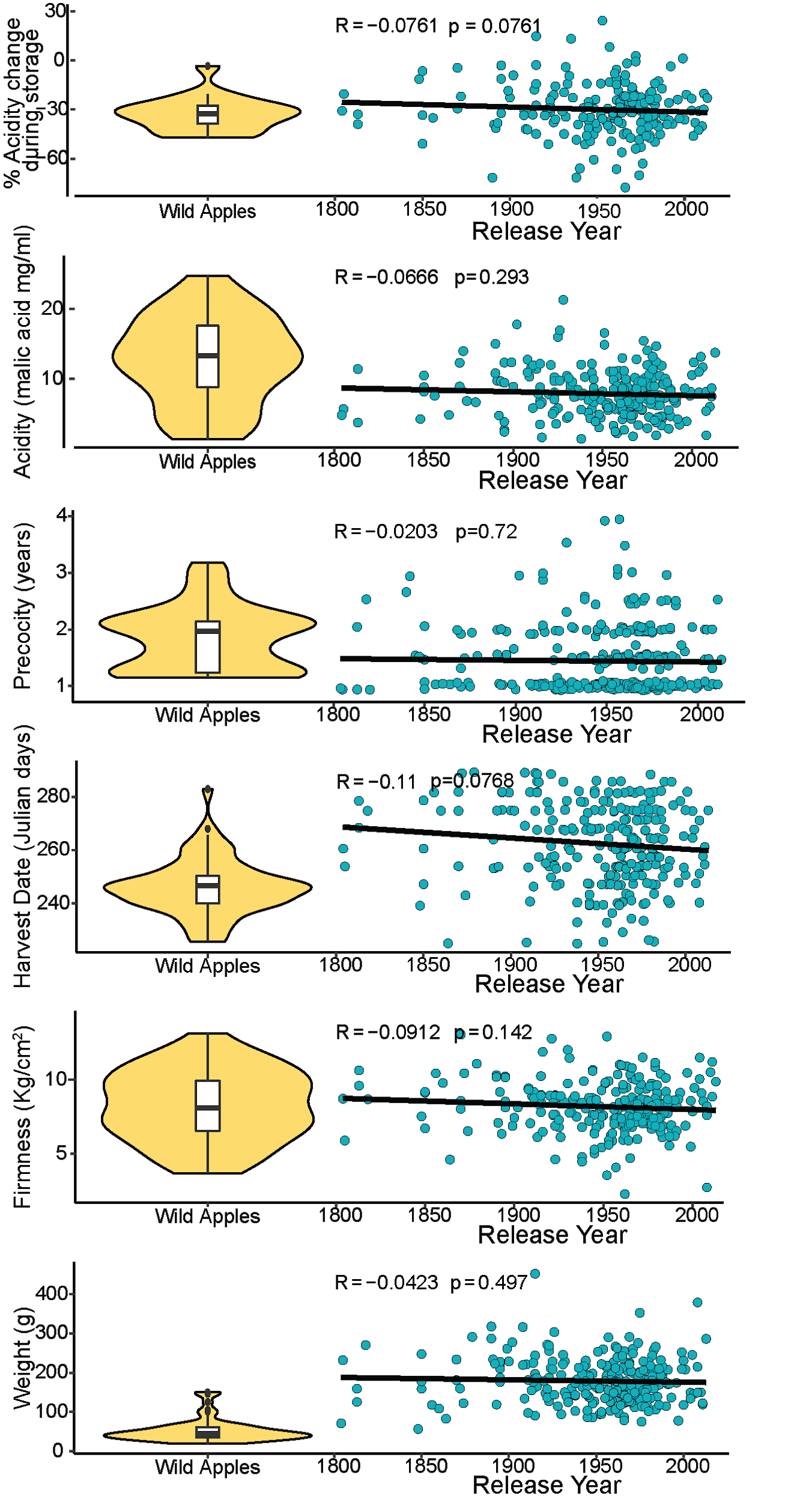


**S1 Fig. Phenotypes of cultivated apples as a function of their release year with a comparison to the ancestral state. Phenotypes include acidity change during storage, acidity, precocity, harvest date, firmness, and weight.** Cultivated apple scores for each phenotype are shown in blue, and the ancestral state of each phenotype is represented in yellow as a density distribution of values from *M. sieversii*. The R and p values from a Pearson correlation between phenotypic values and release year are shown within each scatter plot.
