# Supplementary material for: Phenotypic divergence between the cultivated apple (Malus domestica) and its primary wild progenitor (Malus sieversii)
Source: PLoS One. 2022 Mar 23;17(3):e0250751. doi: 10.1371/journal.pone.0250751 (PMC8942233; doi:10.1371/journal.pone.0250751)
Supplement: Attachment — Submitted filename: Reviewer Report -.docx [file pone.0250751.s002.docx]

**Reviewer Report**

**Manuscript Number:** PONE-D-21-11607

**Title:** Phenotypic divergence between the cultivated apple (*Malus domestica*) and its primary

wild progenitor (*Malus sieversii*)

**Article Type:** Original Paper

**Observations:**

**Introduction –**

The authors have written introduction very nicely with covering the importance of genetic variation with respect to cultivated and wild apple progenitor.

**Materials and Methods –**

1. This section has been written systematically.
2. Please specify the number of apple accessions evaluated for ten phenotypes. Is the present study involving data from 1119 ABC.

**Results**

1. The results obtained by the authors are very interesting.
2. Results indicated the correlation of selected trait in different accessions with respect to year to varietal release and phenological stages.

**Discussion and Conclusion**

The present research is justified with good sources and presented in a well form.

**Overall Recommendation –** Recommended for publication.
