# Supplementary material for: Phenotypic divergence between the cultivated apple (Malus domestica) and its primary wild progenitor (Malus sieversii)
Source: PLoS One. 2022 Mar 23;17(3):e0250751. doi: 10.1371/journal.pone.0250751 (PMC8942233; doi:10.1371/journal.pone.0250751)
Supplement: Attachment — Submitted filename: 20210604_reviewer_responses.docx [file pone.0250751.s003.docx]

Dear Editor,

We thank the reviewers for their comments and suggestions and have revised the manuscript accordingly. Our detailed responses can be found below in red.

Best regards,

Sean Myles

Reviewer #1: Comments:

The article is well structured and nicely organized. It explores the phenotypic divergence between the cultivated apple and its primary wild progenitor. Authors have shown that the cultivated apple has lower acidity and less phenolic content than the wild one. The study has provided an in-depth coverage for variation in fruit and phenology across both species which may help in improving future apple cultivars.

The article could be accepted for publication if following minor revisions are incorporated:

In discussion part, add a holistic approach including phenomics, genomics and metabolomics, which could be utilized for apple improvement programs.

We have revised the discussion to include the folloowing [LINE 276]: “Future holistic evaluations, including a combination of genomic, metabolomic and transcriptomic analyses, will help further assess the degree to which the apple’s wild relatives may contribute to improving apple cultivar development.”

Correct the grammatical mistakes and sentence making. It will be better if help of some native English speaker could be taken.

We welcome the reviewer to point out grammatical or orthographical errors in the manuscript and will happily correct them. However, the manuscript has been reviewed by numerous fluent English speakers and no mistakes have been detected.

References need thorough cross-checking. Please check for journal abbreviations. Provide wherever applicable and avoid unnecessary ones, if not available.

We have reviewed the references and will follow any instructions related to references from the final proof editor as well upon publications.

Reviewer #2: Observations:

Introduction –

The authors have written introduction very nicely with covering the importance of genetic variation with respect to cultivated and wild apple progenitor.

Materials and Methods –

1.This section has been written systematically.

2.Please specify the number of apple accessions evaluated for ten phenotypes. Is the present study involving data from 1119 ABC.

We have added a sentence to the very beginning of the results section [Line 120] as follows: “Sample sizes across 10 phenotypes ranged from 9-76 and 399-797 for wild and cultivated apples, respectively, and are specified in Table 1.”

Results

1.The results obtained by the authors are very interesting.

2.Results indicated the correlation of selected trait in different accessions with respect to year to varietal release and phenological stages.

Discussion and Conclusion

The present research is justified with good sources and presented in a well form.

Overall Recommendation – Recommended for publication
